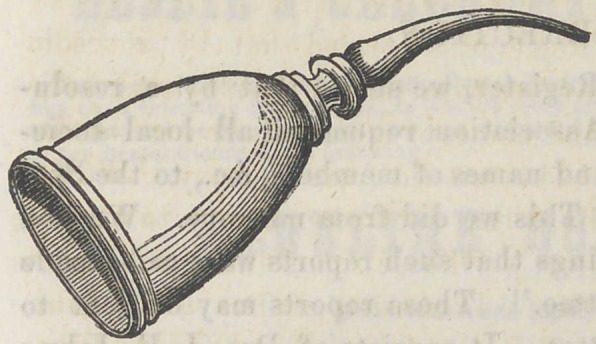# An Extension Thimble

**Published:** 1863-06

**Authors:** 


### AN EXTENSION THIMBLE.

The accompanying cut represents an extension which we have had made, to be used upon the index or middle finger of the left hand. It is employed to aid in holding the napkin, paper, spunk or whatever may be used to prevent the encroachment of saliva. The point of this instrument can extend into the mouth when the finger either on account of its size, or for want of length, cannot go. It may also be used occasionally to hold down a piece of gold till it is made fast in the proper position. We find it so frequently applicable that we would hardly know how to get along without it. In filling the teeth of the left side both above and below it is decidedly advantageous. It will reach over and draw the napkin up firmly against the lingual sides of the teeth. There are points of two or three different forms and sizes which may be attached, as there is a screw joint at the collis. Any worker in precious metals can make them. T.

## Figures and Tables

**Figure f1:**